# Effect of human serum albumin on clinical outcomes in pediatric patients undergoing gastrointestinal surgery

**DOI:** 10.3389/fped.2025.1590586

**Published:** 2025-07-16

**Authors:** Ping Li, Mi Zhou, Da-Yu Chen, Ya-Kun Liu, Feng Liu, Yong-Gen Xu, Jian Wang, Huan Gui

**Affiliations:** ^1^ Department of Clinical Nutrition, Children’s Hospital of Soochow University, Suzhou, China; ^2^ Department of Pharmacy, Children’s Hospital of Soochow University, Suzhou, China; ^3^ Department of Pharmacy, Nanjing Drum Tower Hospital, Nanjing, China; ^4^ Department of General Surgery, Children’s Hospital of Soochow University, Suzhou, China; ^5^ Surgical Intensive Care Unit, Children’s Hospital of Soochow University, Suzhou, China

**Keywords:** human serum albumin, clinical outcome, pediatrics, gastrointestinal surgery, propensity score matching

## Abstract

**Purpose:**

This study aims to evaluate the effectiveness of administering 20% human serum albumin (HSA) on short-term clinical outcomes in pediatric patients undergoing enteric anastomosis, with a specific focus on postoperative hospital stay (PHS), postoperative fasting duration (PFD), and the incidence of postoperative complications (PCs).

**Methods:**

This was a single-center, retrospective cohort study. Patients aged between 1 month and 18 years who underwent simple intestinal anastomosis were included. Comprehensive data, including patient demographics, prescribed medications, laboratory test results, and surgical records, were meticulously extracted from electronic patient dossiers. The primary endpoint was PHS. The second endpoint included PFD and PCs. Since this was a retrospective cohort study, we used propensity score matching (PSM) to balance different variables. The efficacy of 20% HSA on clinical outcomes was assessed by univariate and multivariate logistic regression analyses.

**Results:**

Among a cohort of 242 patients, 67 (27.69%) were administered 20% HSA for over 2 days during the early postoperative stage. A dose-related pattern of HSA efficacy on clinical outcomes was observed in the PSM cohort. After adjustment, HSA overuse was identified as an independent risk factor for prolonged PHS and a higher complication incidence, with odds ratios of 6.56 [95% confidence interval (CI): 2.12–20.32] and 5.14 (95% CI: 1.21–21.83), respectively.

**Conclusions:**

Overuse of 20% HSA in the early postoperative stage does not contribute to improved clinical outcomes in pediatric patients undergoing gastrointestinal surgery.

## 1 Introduction

Albumin, a principal protein in the human body, exerts a pivotal influence on numerous physiological mechanisms, such as the transport of various molecules such as electrolytes and pharmacological agents, engagement in oxidation–reduction reactions, and the transduction of biological signals (1). Clinically, albumin is frequently administered to augment intravascular volume or sustain colloid oncotic pressure (COP), thereby ensuring equilibrium between intravascular and extravascular spaces (2). In addition, serum albumin levels often decrease after major surgery (3–5), and it is well known that the degree of decrease in perioperative albumin is an independent risk factor for adverse outcomes (6, 7). Thus, albumin is usually prescribed to temporarily manage acute hypoalbuminemia in the surgical setting. However, the benefit of postoperative albumin administration on clinical outcomes remains inconclusive.

Recent findings from randomized controlled trials (RCTs) and retrospective studies on albumin use in patients undergoing cardiac surgery have been inconsistent (8, 9). While albumin is increasingly being used in non-cardiac surgeries worldwide, data on its postoperative efficacy in children undergoing major abdominal surgery remain scarce. Early studies have also indicated a dose-dependent relationship between albumin therapy and complication rates (10), indicating that excessive albumin use may be harmful. However, the boundary between excessive and non-excessive albumin use remains obscure in children. Given the dynamic shifts in fluid balance and inflammatory status during the early postoperative period, we defined human serum albumin (HSA) overuse as administration for ≥2 consecutive days to distinguish necessary early-phase support from potentially harmful prolonged use.

This study evaluates the effectiveness of albumin administration on short-term clinical outcomes in pediatric patients undergoing enteric anastomosis surgery, with a focus on postoperative hospital stay (PHS), postoperative fasting duration (PFD), and the incidence of postoperative complications (PCs). This research provides valuable insights into the use of albumin in pediatric patients, an area with limited existing data.

## 2 Materials and methods

### 2.1 Patients and materials

This was a retrospective, single-center, propensity score-matched study. Patients aged 1 month to 18 years who underwent single intestinal anastomosis at the Children's Hospital of Soochow University from June 2021 to September 2024 were identified from the computerized hospital information system (HIS). Exclusion criteria included neonates, patients who underwent non-digestive tract procedures, hepatobiliary and upper gastrointestinal tract surgeries, or had incomplete medical records. Preoperative bowel preparation, fasting regimen, and preoperative medication followed the ruling standard protocols. Patients were divided into two groups based on the frequency of 20% HSA administration within 72 h after surgery. The HSA overuse group was defined as patients who received HSA for two or more consecutive days during the observation period. Patients who received no HSA or received HSA for only 1 day after surgery were classified into the non-overuse group. This threshold was selected based on clinical observations and existing evidence on pathophysiological changes during the acute postoperative phase, which typically lasts 24–72 h. During this period, increased inflammatory responses and heightened capillary permeability may justify temporary albumin supplementation to maintain circulatory stability. However, prolonged use beyond the immediate postoperative phase may increase the risk of fluid overload, tissue edema, and impaired healing.

### 2.2 Endpoints

The primary endpoint was PHS, calculated from the conclusion of surgery to the time of discharge, as it impacts the overall burden on the child and their family. Secondary endpoints included PFD, defined as the period from surgery completion to the resumption of unhampered oral intake of liquid food. This parameter is a crucial prognostic factor and has not been extensively studied in pediatric patients. Secondary endpoints also included the prevalence of PCs observed during hospitalization. The monitored PCs included infections (such as pulmonary and surgical site infections), fistulae (identified via CT scan and/or characteristics of the drainage material), hemorrhage, abdominal distension, diarrhea, and intestinal obstruction, all of which were significant concerns in pediatric surgery.

### 2.3 Data collection

Comprehensive data, including patient demographics, prescribed medications, laboratory test results, and surgical records, were meticulously extracted from electronic patient dossiers. Growth and development levels were assessed using the Z-score curve charts, referencing children's height and weight against the 2006 World Health Organization (WHO) growth standards for children aged 0–5 years and the 2007 WHO standards for those aged 5–19 years. The weight-for-age Z-score (WAZ) was utilized as the growth and development indicator for children aged 0–5 years, while the body mass index (BMI)-for-age Z-score (BAZ) was applied for those aged 5–18 years. WAZ and BAZ values within the range of −2 to 2 were classified as indicative of normal growth and development, whereas values outside this range were considered reflective of abnormal growth patterns. An independent reviewer rigorously verified the raw data, including perioperative liquid prescriptions, primary and secondary endpoints, and the occurrence of postoperative complications, to ensure accuracy and reliability.

### 2.4 Statistical analysis

Continuous variables were presented as means ± standard deviations (SD) for normally distributed data or as medians with interquartile ranges (IQR) for non-normally distributed data. Categorical variables were described using frequencies and percentages. Before regression analysis, all data were inspected for missing values. Among the potential factors, the proportion of missing data was 5.78% for baseline serum albumin and 3.31% for baseline serum hemoglobin. To include these data in the subsequent analysis, mean imputation was carried out.

To strengthen the robustness of our findings, we employed a three-stage analytical strategy. First, we conducted univariate and multivariate logistic regression analyses on the full cohort to identify potential risk factors associated with clinical outcomes. During these analyses, we observed baseline imbalances between the HSA overuse and non-overuse groups. To better address potential confounding arising from this imbalance, we conducted propensity score matching (PSM) using covariates associated with the outcomes but not interacting with HSA use, including age, baseline serum albumin levels, and surgery duration. We matched the two groups in a 1:1 ratio using a caliper width of 0.2. A standardized mean difference (SMD) of <0.2 was considered indicative of adequate balance. Data processing and analysis were performed using R version 4.4.0 (24 April 2024), along with Zstats 1.0 (https://www.zstats.net). Following PSM, we re-analyzed the matched cohort using logistic regression to evaluate the association between HSA overuse and clinical outcomes. Odds ratios (ORs) with 95% confidence intervals (CIs) and *P*-values were reported. A *P-*value of <0.05 was considered statistically significant. Statistical analysis was performed using SPSS for Windows, version 23.0 (SPSS Inc., Chicago, IL, USA)

## 3 Results

### 3.1 Patient characteristics

A total of 242 patients were included in the study. The inclusion and exclusion processes are summarized in a flowchart (see Supplementary data Figure S1). All patients achieved either recovery or improvement of their primary disease. As shown in Table 1, the median age was 67.5 months. Male children accounted for 47.50% of the total study population, and 84.71% of patients exhibited normal growth and development. A total of 214 patients were diagnosed with intestinal tract-related conditions, including Meckel's diverticulum, intussusception, structural malformations, perforation, obstruction, necrosis, appendicitis, and closure of ileostomy. Twenty-eight patients were diagnosed with extraintestinal tract-related conditions, including peritoneal tumors/cysts and intestinal foreign bodies (the detailed etiological distribution of the patients is summarized in Supplementary data Table S1). The mean baseline serum albumin level was 45.75 ± 4.69 g L^−1^, and the mean baseline serum hemoglobin level was 136.50 ± 21.79 g L^−1^. The duration of surgery was no less than 150 min in 60 patients. Most patients in the cohort experienced blood loss of less than 10% of the estimated blood volume (EBV), with only nine patients exceeding this threshold. The crystalloid infusion volume was standardized according to body weight. The average volume of crystalloid infusion was 85.54 ± 41.98 mL kg^−1^ day^−1^ during the first 24 h after surgery and 68.66 ± 31.33 mL kg^−1^ day^−1^ over the first 72 h after surgery. A total of 67 patients, accounting for 27.69% of total patients, were over-prescribed 20% HSA after surgery. Nearly 54.00% of all children received total parenteral nutrition (TPN) therapy postoperatively. Plasma or red blood cell suspensions were administered to 24.38% of all patients after surgery. Postoperative opioids were used for analgesia in 24 patients. Opioid use included fentanyl and/or remifentanil, which were infused via continuous infusion pumps. Regarding clinical outcomes, the median PHS was 7.46 days, with 54.55% of patients experiencing prolonged PHS (greater than 7 days). The median PFD was 3.05 days, and 50% of patients had prolonged PFD (greater than 3 days). A total of 36 complication events were recorded, accounting for up to 14.88% of the total study population.

**Table 1:** Patient characteristics.

Variables	Overall population (*n* = 242)
Baseline characteristics	
Age (months), median (Q1, Q3)	67.5 (25, 111.5)
Male sex, *n* (%)	115 (47.50%)
Normal in growth and development, *n* (%)	205 (84.71%)
Major diagnosis	
Intestine tract-related diseases	214 (88.43%)
Extraintestinal-related diseases	28 (11.57%)
Baseline serum albumin (g L^−1^)	45.75 ± 4.69
Baseline serum hemoglobin (g L^−1^)	136.5 ± 21.79
Duration of surgery (min)	110 (80, 150)
Duration of surgery (≥150 min)	60 (24.79%)
Blood loss during the procedure (≥10% EBV)	9 (3.72%)
Crystalloid V_24h_ (mL kg^−1^ day^−1^)	85.53 ± 41.98
Crystalloid V_72h_ (mL kg^−1^ day^−1^)	68.66 ± 31.33
HSA overuse	67 (27.69%)
Postoperative TPN use	131 (54.13%)
Postoperative blood transfusion	59 (24.38%)
Postoperative opioid use	24 (9.92%)
Clinical outcomes	
PHS (days), median (Q1, Q3)	7.46 (6.50, 8.94)
PHS (>7 days), *n* (%)	132 (54.55)
PFD (days), median (Q1, Q3)	3.05 (3.00, 4.29)
PFD (>3 days), *n* (%)	121 (50.00)
PC, *n* (%)	36 (14.88)
Infection	20 (8.26)
Hemorrhage	7 (2.89)
Abdominal distension	4 (1.65)
Diarrhea	2 (0.83)
Ileus	2 (0.83)
Fistula	1 (0.41)

HSA, human serum albumin; TPN, total parenteral nutrition; V_24h_, volume within 24 h after surgery; V_72h_, volume within 72 h after surgery; PFD, postoperative fasting duration; PHS, postoperative hospital stay; PC, postoperative complication.

Data are presented as means ± SDs, medians (interquartile ranges), or numbers (percentages).

### 3.2 Propensity score-matching analysis

Table 2 summarizes the characteristics of the HSA non-overuse and overuse groups before and after PSM. Before PSM, 67 patients (27.68%) received albumin for at least two consecutive postoperative days. Significant imbalances were found between the non-overuse and overuse groups in terms of age, baseline serum albumin levels, surgery duration, bleeding volume, crystalloid infusion volume, blood transfusion, TPN use, and opioid use. We performed propensity score matching on age, baseline serum albumin levels, and surgery duration. After matching, 49 patients were included in each group. Most confounding variables achieved balance between the groups; however, differences persisted in crystalloid volume within 72 h (V_72h_), blood transfusion, TPN use, and opioid use. These variables were closely linked to albumin use. We conducted a multivariate analysis to evaluate their impact on clinical outcomes.

**Table 2:** Comparison of baseline characteristics between HSA overuse and no-overuse groups before and after PSM.

Variable	Baseline characteristics			Propensity score-matched baseline		
	Non-overuse HSA (*n* = 175)	Overuse HSA (*n* = 67)	*P*	Non-overuse HSA (*n* = 49)	Overuse HSA (*n* = 49)	*P*
Sex			0.534			0.685
Female	94 (53.71)	33 (49.25)		28 (57.14)	26 (53.06)	
Male	81 (46.29)	34 (50.75)		21 (42.86)	23 (46.94)	
Age (years)			0.002			0.686
>5	110 (62.86)	27 (40.30)		25 (51.02)	23 (46.94)	
≤5	65 (37.14)	40 (59.70)		24 (48.98)	26 (53.06)	
Growth and development			0.271			1.000
Normal	151 (86.29)	54 (80.60)		41 (83.67)	41 (83.67)	
Abnormal	24 (13.71)	13 (19.40)		8 (16.33)	8 (16.33)	
Major diagnosis			0.704			0.400
Intestine tract-related	154 (88.00)	60 (89.55)		40 (81.63)	43 (87.76)	
Extraintestinal tract-related	21 (12.00)	7 (10.45)		9 (18.37)	6 (12.24)	
Baseline albumin (g L^−1^)	44.10 (41.48, 46.50)	42.15 (37.50, 45.22)	0.003	43.20 (38.80, 45.50)	42.40 (39.20, 45.40)	0.980
Baseline hemoglobin (g L^−1^)	122.00 (105.50, 131.00)	119.00 (100.75, 129.25)	0.369	118.50 (99.75, 126.00)	121.00 (102.25, 135.25)	0.463
Surgery duration (min)			<0.001			0.825
Under 150	145 (82.86)	37 (55.22)		35 (71.43)	34 (69.39)	
Over 150	30 (17.14)	30 (44.78)		14 (28.57)	15 (30.61)	
Bleeding (mL)			0.022			0.359
<10% EBV	172 (98.29)	61 (91.04)		48 (97.96)	45 (91.84)	
≥10% EBV	3 (1.71)	6 (8.96)		1 (2.04)	4 (8.16)	
Crystalloid V_24h_ (mL kg^−1^ day^−1^)	89.05 ± 38.10	108.73 ± 48.22	0.003	90.88 (56.09, 113.11)	110.57 (65.16, 134.74)	0.102
Crystalloid V_72h_ (mL kg^−1^ day^−1^)	74.79 ± 27.41	94.65 ± 36.26	<0.001	72.46 (51.40, 99.53)	98.48 (64.08, 119.74)	0.016
Blood transfusion			<0.001			<0.001
No	156 (89.14)	27 (40.30)		42 (85.71)	20 (40.82)	
Yes	19 (10.86)	40 (59.70)		7 (14.29)	29 (59.18)	
TPN use			<0.001			0.002
No	96 (54.86)	15 (22.39)		28 (57.14)	13 (26.53)	
Yes	79 (45.14)	52 (77.61)		21 (42.86)	36 (73.47)	
Opioid use			<0.001			0.004
No	168 (96.00)	50 (74.63)		48 (97.96)	39 (79.59)	
Yes	7 (4.00)	17 (25.37)		1 (2.04)	10 (20.41)	

HSA, human serum albumin; PSM, propensity score matching; EBV, estimated blood volume; TPN, total parenteral nutrition; V_24h_, volume within 24 h after surgery; V_72h_, volume within 72 h after surgery.

Data are presented as means ± SDs, medians (interquartile ranges), or numbers (percentages).

### 3.3 Exploration of dose–response relationship of HSA use on PHS in the PSM cohort

**Figure 1:**
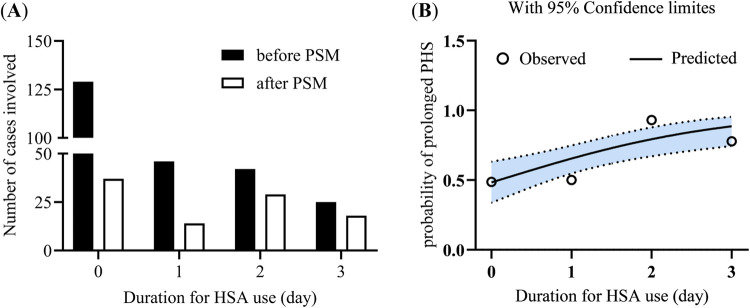
Frequency distribution of HSA use and its ability to predict prolonged PHS. **(A)** Frequency distribution of patients according to the duration of HSA use. **(B)** Predicted probability (line, with 95% confidence limits) vs. observed probability (circle) of prolonged PHS across varying durations of HSA use. HSA, human serum albumin; PSM, propensity score matching; PHS, postoperative hospital stay.

Figure 1A compares the frequency distribution of different administration days of HSA use between the original and PSM populations. The number of patients who received no HSA decreased dramatically from 129 to 37. Of the 46 patients who received HSA for only 1 day, 14 were included in the PSM cohort. Ultimately, the ratio between the HSA overuse and non-overuse groups reached 1:1, with each group comprising 49 patients. Figure 1B illustrates the observed (circles) and logistic model-predicted (line) probabilities of prolonged PHS according to the number of days of HSA use. In general, the probability of prolonged PHS increases with the number of days of HSA use. Overuse of HSA, such as administration for 2 or 3 days, is associated with a significantly higher predicted probability of prolonged PHS. It was observed that 93.10% (27 out of 29) of patients who received HSA for 2 days and 77.78% (14 out of 18) of those who received HSA for 3 days experienced prolonged PHS.

### 3.4 Risk factor evaluation for clinical outcomes before PSM and after PSM

**Figure 2:**
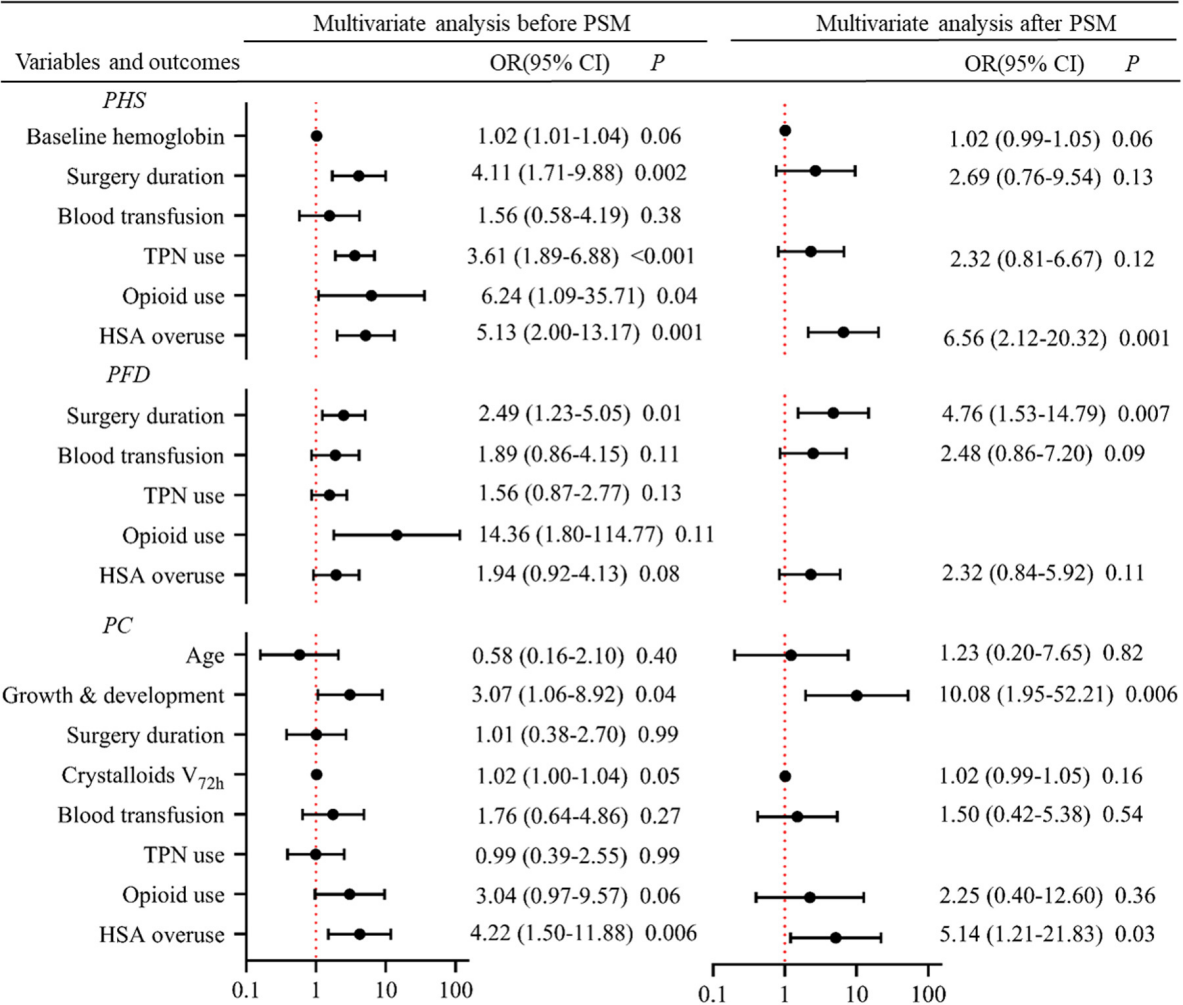
Risk factor analysis for clinical outcomes before and after PSM using multivariate logistic regression. Each line represents the 95% CI for the effect of a covariate. The black dot indicates the estimated effect size (OR). The *X*-axis is plotted on a log_10_ scale. The vertical red dashed line at OR = 1 represents no effect. An OR > 1 means that the covariate is a risk factor. HSA, human serum albumin; PSM, propensity score matching; PHS, postoperative hospital stay; PFD, postoperative fasting duration; PC, postoperative complications; TPN, total parenteral nutrition.

Univariate analysis revealed that baseline hemoglobin levels, surgery duration exceeding 150 min, TPN use, and HSA overuse were associated with prolonged PHS of more than 7 days, both before and after PSM (see Supplementary data Table S2 for details). Multivariate analysis results, as depicted in Figure 2, indicated that HSA overuse was an independent risk factor for prolonged PHS exceeding 7 days, irrespective of whether the analysis was conducted before or after PSM. In the pre-PSM population, the adjusted OR for HSA use was 5.13 (95% CI: 2.00–13.17, *P* = 0.001). This risk further increased after PSM, with an adjusted OR of 6.56 (95% CI: 2.12–20.32, *P* = 0.001).

Univariate analysis showed that prolonged PFD was consistently associated with extended surgery duration, blood transfusion, and postoperative HSA administration in both populations, both before and after PSM (see Supplementary data Table S3). However, in the multivariate analysis, the impact of HSA administration was diminished. Surgery duration emerged as the sole consistent independent risk factor for prolonged PFD, with an OR of 2.49 (95% CI: 1.23–5.05, *P* = 0.01) before PSM and 4.76 (95% CI: 1.53–14.79, *P* = 0.007) after PSM.

Univariate analysis identified numerous potential risk factors associated with PC incidence , including younger age, abnormal growth and development, prolonged surgery duration (>150 min), excessive crystalloid volume within 72 h postoperation, blood transfusion, TPN use, opioid treatment, and HSA overuse (see Supplementary data Table S4). After adjustment in the multivariate regression analysis, abnormal growth and development and HSA administration were identified as independent risk factors for PC incidence. In the PSM population, the OR for the impact of growth and development on PC incidence was 10.08 (95% CI: 1.95–52.21, *P* = 0.006), which is quite remarkable. In the same population, the OR for the impact of HSA administration on PC incidence was 5.14 (95% CI: 1.21–21.83, *P* = 0.03).

## 4 Discussion

In this observational study of pediatric patients who underwent single enterostomy, we employed PSM and found that the overuse of 20% HSA postoperatively was associated with adverse clinical outcomes. Specifically, compared to patients who had no exposure or a single early exposure to 20% HSA within 72 h after surgery, those who received HSA for more than two consecutive days postsurgery were at a higher risk for prolonged PHS and increased incidence of PCs, with ORs of 6.56 for PHS and 5.14 for PC. We also noted that the effect of albumin appeared to follow a dose-dependent trend. However, it remains unclear whether the increased frequency of albumin transfusion reflects the surgeon's subjective assessment of a heightened risk for postoperative complications in certain patients or if the clinical outcomes are directly attributable to the increased frequency of albumin administration. These findings are particularly significant in pediatric surgery, as they underscore the importance of careful consideration of HSA administration in pediatric patients undergoing intestinal surgery.

Current evidence directly linking HSA use to adverse clinical outcomes is limited, particularly in pediatric populations. Clinically, 20% albumin is often administered to recruit interstitial fluid and increase plasma volume (11). However, the implications of this practice in pediatric surgery remain unclear due to inconsistent findings across studies, likely arising from diverse patient populations and varying albumin dosing regimens. In pediatric cardiac surgery research, albumin has been added to the cardiopulmonary bypass prime fluid as an alternative to crystalloids; however, conclusions regarding its clinical application value have been inconsistent across studies (8, 12, 13). Few studies have examined the association between short-term albumin use and clinical outcomes in children undergoing intestinal surgery. However, a retrospective analysis of adults undergoing abdominal surgery found that intraoperative administration of 20% albumin was significantly associated with a higher risk of prolonged hospital stays, regardless of whether the data were unadjusted or adjusted (14). In this study, the dosage of 20% albumin ranged from 100 to over 500 mL, with a significant proportion of patients undergoing colorectal and gynecological surgeries. In an adult RCT involving moderate to major gastrointestinal surgery, participants in the albumin group received 100 mL of 20% human albumin daily from the day of surgery through postoperative day 2 (totaling 300 mL). No significant differences were observed in PHS or PC rates between the albumin and saline groups. However, the incidence of adverse events was higher in the albumin group. Notably, early postoperative exogenous albumin supplementation was ineffective in correcting hypoalbuminemia in this study (15). In our study, all children underwent simple intestinal anastomosis for intestinal-related diseases, and albumin was administered postoperatively at varying dosages. We utilized PSM to balance baseline confounders between groups. Consistent with findings in adult populations, we observed that 20% albumin administered was significantly associated with prolonged PHS in both unadjusted and adjusted analyses. It appears that the correlation between albumin use and adverse short-term outcomes in abdominal surgery may also apply to pediatric patients.

Few studies have established a causal link between perioperative albumin exposure and adverse outcomes. Postoperative intestinal edema can significantly hinder the recovery of gastrointestinal function after surgery (16). It is well-established that both intraoperative and postoperative fluid overload can contribute to tissue edema, adversely affecting the healing of intestinal anastomoses (17). A recent clinical trial highlighted the prevalence of over-hydration in patients undergoing acute high-risk abdominal surgery, such as those with intestinal obstruction and anastomotic leakage (18). Excessive infusion of albumin, when combined with over-hydration, can lead to circulatory overload, reduced vascular colloid osmotic pressure, increased capillary hydrostatic pressure, and ultimately fluid leakage into the interstitial space, leading to edema (19). There is no unified standard for fluid resuscitation in pediatric surgical patients, making it challenging to determine optimal fluid management strategies. In our study, we found that both the crystalloid volume administered within 72 h after surgery and excessive HSA use were associated with a higher incidence of complications, as demonstrated by univariate logistic regression analysis. However, we did not specifically assess the occurrence of fluid overload at specific crystalloid volumes. In our multivariate analysis, the impact of crystalloid volume on PC incidence was less significant, while HSA use emerged as a very strong independent risk factor for PC. These findings highlight the importance of judicious HSA use during postoperative fluid resuscitation.

Surgical procedures typically lead to endothelial damage and trigger a localized or systemic inflammatory response, resulting in decreased vascular permeability and potential albumin leakage at the site (20). This phenomenon is further complicated by elevated levels of C-reactive protein (CRP), a key marker of inflammation, which can disrupt endothelial function by directly disrupting the glycocalyx (21). While glycocalyx shedding and capillary leakage have been mostly reported in cardiac surgery and in severely ill patients (22, 23), they are less commonly observed in cases of hypervolemia (24) and prolonged (6 h) abdominal surgery (25). Unfortunately, due to the lack of comprehensive data on CRP levels and evidence of inflammation, we could not conclusively associate HSA extravasation with these processes in the present study.

For the first time, our study identified early overuse of HSA postsurgery as a potential risk factor for prolonged fasting time in children. Extended postoperative fasting is known to increase the risk of complications (26, 27). This finding is supported by various studies in both adult and pediatric populations, which have shown the benefits of early initiation of enteral nutrition following intestinal surgery (28). Although the impact of HSA on PFD was reduced by other cofounders in the multivariate analysis, it remains a plausible contributing factor to prolonged PHS and higher PC incidence. Our study identified surgical procedures lasting longer than 150 min as an independent factor associated with prolonged postoperative fasting time. This is likely due to the increased complexity and duration of such procedures, which often necessitate larger doses of opioid analgesics; these medications could impact the recovery of gastrointestinal function in postsurgical patients (29), often leading to extended postoperative fasting times. HSA is known for its ability to bind to a wide range of drugs, including opioid analgesics (30). This binding capacity can significantly influence the pharmacokinetics of these drugs, particularly in the context of hypoproteinemia (31, 32). Based on this understanding, it makes sense to hypothesize that HSA, by binding to opioid analgesics, may potentially delay their metabolism and excretion, leading to prolonged suppression of gastrointestinal motility.

This study has several limitations. First, its retrospective design inherently limits causal inference and may introduce selection and information biases. Second, although serum albumin was measured preoperatively, we did not assess other key acute-phase biochemical markers, such as preserum albumin and C-reactive protein, which could have provided a more comprehensive understanding of the inflammatory and nutritional status of patients before and after surgery. Third, the cohort included a wide range of diagnoses, introducing clinical heterogeneity despite all patients undergoing a single type of intestinal anastomosis. Finally, although we used multivariate analysis and propensity score matching to adjust for confounders, residual confounding and unmeasured variables may still have influenced the outcomes. Further prospective studies are needed to confirm these conclusions with greater certainty.

In conclusion, our study suggests that excessive early postoperative administration of HSA does not improve clinical outcomes in pediatric patients undergoing gastrointestinal surgery. Given the high cost of albumin, its routine use may not be cost-effective. We recommend that clinicians exercise caution when prescribing albumin for this patient population, assessing each case individually for potential risks and benefits. The decision to administer albumin should be individualized, guided by the specific needs of each child and aligned with the latest evidence and guidelines in pediatric surgery.

## Data Availability

The raw data supporting the conclusions of this article will be made available by the authors without undue reservation.
