# Supplementary figures and images for: Effect of human serum albumin on clinical outcomes in pediatric patients undergoing gastrointestinal surgery
Source: Front Pediatr. 2025 Jul 16;13:1590586. doi: 10.3389/fped.2025.1590586 (PMC12307337; doi:10.3389/fped.2025.1590586)

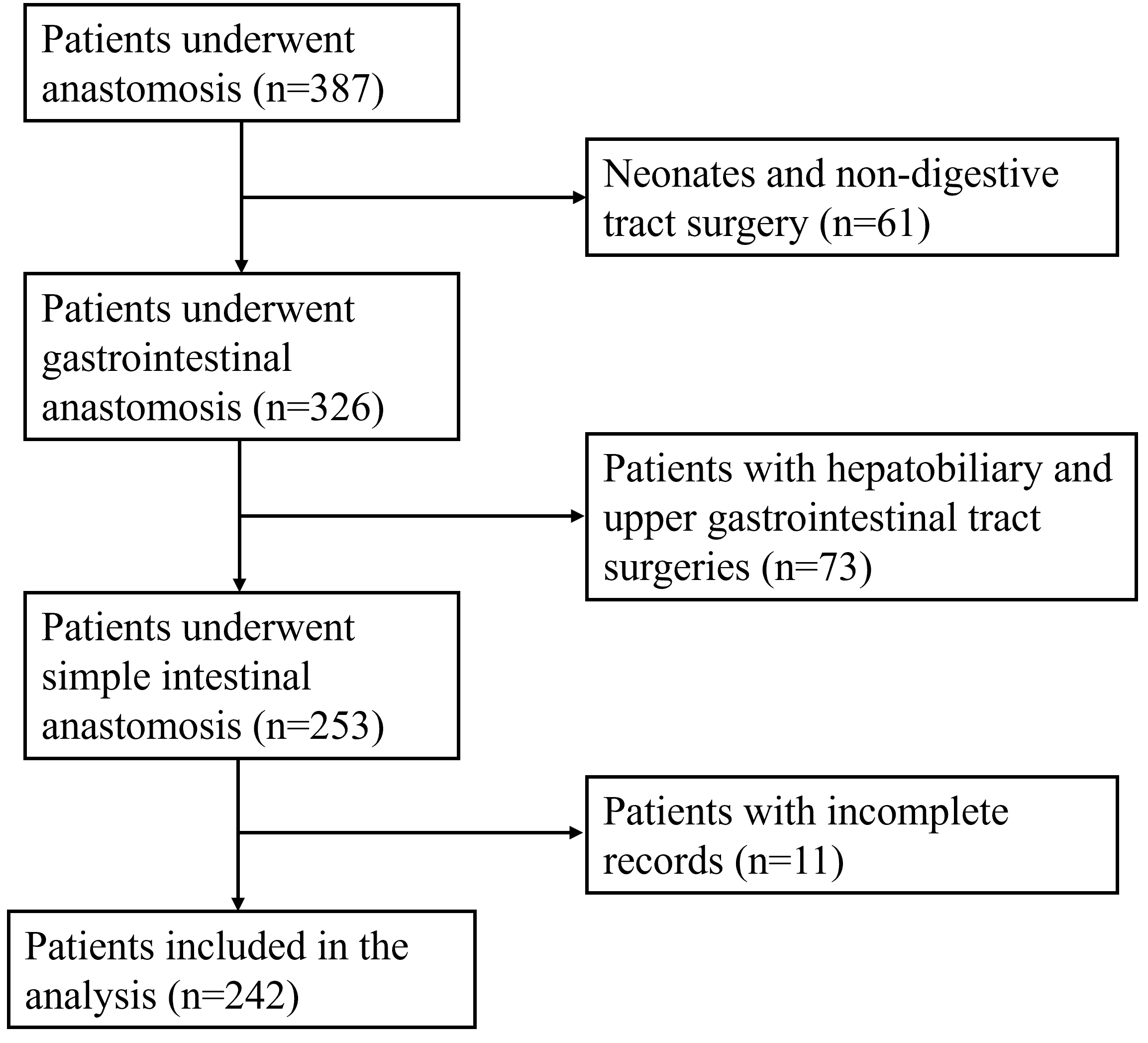

Supplement: Supplementary file 1 [file Image1.tif]
