# Supplementary material for: Effect of human serum albumin on clinical outcomes in pediatric patients undergoing gastrointestinal surgery
Source: Front Pediatr. 2025 Jul 16;13:1590586. doi: 10.3389/fped.2025.1590586 (PMC12307337; doi:10.3389/fped.2025.1590586)
Supplement: Supplementary file 2 [file Supplementaryfile1.docx]

**Supplemental data Table S1**. **Etiology distribution of enrolled patients**

| **Etiology** | **Number** | **Percentage** |
| --- | --- | --- |
| **Intestine tract related diseases (n=214)** |  |  |
| Meckel’s diverticulum | 117 | 48.35% |
| intussusception | 20 | 8.26% |
| structural malformation | 18 | 7.44% |
| perforation | 18 | 7.44% |
| obstruction | 17 | 7.02% |
| necrosis | 6 | 2.48% |
| appendicitis | 9 | 3.72% |
| close the ileostomy | 9 | 3.72% |
| **Extra intestinal tract related diseases (n=28)** |  |  |
| Peritoneal tumor/cyst | 16 | 6.61% |
| Intestinal foreign body | 12 | 4.96% |
