# Supplementary material for: Effect of human serum albumin on clinical outcomes in pediatric patients undergoing gastrointestinal surgery
Source: Front Pediatr. 2025 Jul 16;13:1590586. doi: 10.3389/fped.2025.1590586 (PMC12307337; doi:10.3389/fped.2025.1590586)
Supplement: Supplementary file 3 [file Supplementaryfile2.docx]

**Supplemental data Table S2**. **Risk factor of prolonged PHS before and after PSM by univariate analysis**

| Characteristics | Before PSM | | After PSM | |
| --- | --- | --- | --- | --- |
|  | OR (95% CI) | P | OR (95% CI) | P |
| Baseline hemoglobin | 1.01 (1.00-1.03) | 0.031 | 1.03 (1.00-1.05) | 0.018 |
| Surgery duration | 7.07 (3.28-15.21) | <0.001 | 3.09 (1.05-9.07) | 0.04 |
| Blood transfusion | 3.55 (1.82-6.91) | <0.001 | 2.21 (0.87-15.64) | 0.10 |

| Characteristics | Before PSM | | After PSM | |
| --- | --- | --- | --- | --- |
|  | OR (95% CI) | P | OR (95% CI) | P |
| Baseline hemoglobin | 1.01 (1.00-1.03) | 0.031 | 1.03 (1.00-1.05) | 0.018 |
| Surgery duration | 7.07 (3.28-15.21) | <0.001 | 3.09 (1.05-9.07) | 0.04 |
| Blood transfusion | 3.55 (1.82-6.91) | <0.001 | 2.21 (0.87-15.64) | 0.10 |
| TPN use | 4.19 (2.44-7.18) | <0.001 | 2.93 (1.22-6.99) | 0.016 |
| opioid use | 10.80 (2.48-47.05) | 0.002 | 5.54 (0.68-45.29) | 0.11 |
| HAS overuse | 10.31 (4.64-22.87) | <0.001 | 8.10 (2.92-22.51) | <0.001 |

*HSA* human serum albumin, *PHS* postoperative hospital stay, *PSM* propensity score matching, *TPN* total parenteral nutrition, *OR* odds ratio, *CI* confidence interval
