# Supplementary material for: Effect of human serum albumin on clinical outcomes in pediatric patients undergoing gastrointestinal surgery
Source: Front Pediatr. 2025 Jul 16;13:1590586. doi: 10.3389/fped.2025.1590586 (PMC12307337; doi:10.3389/fped.2025.1590586)
Supplement: Supplementary file 4 [file Supplementaryfile3.docx]

**Supplemental data Table S3**. **Risk factor of prolonged PFD before and after PSM by univariate analysis**

| Characteristics | Before PSM | | After PSM | |
| --- | --- | --- | --- | --- |
|  | OR (95% CI) | P | OR (95% CI) | P |
| Duration of surgery | 3.75 (1.97-7.14) | <0.001 | 3.92 (1.34-11.46) | 0.013 |
| Blood transfusion | 4.04 (2.10-7.78) | <0.001 | 2.88 (1.14-7.32) | 0.026 |
| TPN use | 2.17 (1.30-3.64) | 0.003 | 2.03 (0.88-4.69) | 0.096 |
| opioid use | 28.16 (3.74-212.27) | 0.001 | -- | 0.99 |
| HAS overuse | 4.31 (2.30-8.07) | <0.001 | 2.96 (1.25-6.99) | 0.013 |

*HSA* human serum albumin, *PFD* postoperative fasting duration, *PSM* propensity score matching, *TPN* total parenteral nutrition, *OR* odds ratio, *CI* confidence interval
