# Supplementary material for: Effect of human serum albumin on clinical outcomes in pediatric patients undergoing gastrointestinal surgery
Source: Front Pediatr. 2025 Jul 16;13:1590586. doi: 10.3389/fped.2025.1590586 (PMC12307337; doi:10.3389/fped.2025.1590586)
Supplement: Supplementary file 5 [file Supplementaryfile4.docx]

**Supplemental data Table S4**. **Risk factor of PC before and after PSM by univariate analysis**

| Characteristics | Before PSM | | After PSM | |
| --- | --- | --- | --- | --- |
|  | OR (95% CI) | P | OR (95% CI) | P |
| Age | 2.39 (1.13-5.02) | 0.02 | 2.76 (1.02-7.48) | 0.046 |
| Growth and development | 2.79 (1.20-6.48) | 0.02 | 4.47 (1.45-13.81) | 0.009 |
| Surgery duration | 2.47 (1.16-5.26) | 0.02 | 1.37 (0.51-3.71) | 0.53 |
| Crystalloids V_72h_ | 1.02 (1.01-1.04) | <0.001 | 1.02 (1.01-1.04) | 0.002 |
| Blood transfusion | 6.19 (2.88-17.86) | <0.001 | 4.82 (1.78-13.04) | 0.002 |
| TPN use | 2.27 (1.03-4.97) | 0.04 | 1.90 (0.70-5.14) | 0.21 |
| opioid use | 7.17 (2.88-17.86) | <0.001 | 4.94 (1.35-18.13) | 0.02 |
| HAS overuse | 9.21 (4.10-20.71) | <0.001 | 7.13 (2.21-23.03) | 0.001 |

*HSA* human serum albumin, *PC* postoperative complication, *PSM* propensity score matching, *TPN* total parenteral nutrition, *OR* odds ratio, *CI* confidence interval
